# Inhalation of volatile anesthetics via a laryngeal mask is associated with lower incidence of intraoperative awareness in non-critically ill patients

**DOI:** 10.1371/journal.pone.0186337

**Published:** 2017-10-26

**Authors:** Pei-Jen Kuo, Chia-Ling Lee, Jen-Huang Wang, Shiu-Ying Hsieh, Shian-Che Huang, Chen-Fuh Lam

**Affiliations:** 1 Department of Anesthesiology, Buddhist Tzu-Chi General Hospital, Hualien, Taiwan; 2 Department of Medical Research, Buddhist Tzu-Chi General Hospital, Hualien, Taiwan; 3 Department of Anesthesiology, E-Da Hospital/E-Da Cancer Hospital/I-Shou University, Kaohsiung, Taiwan; Southeast University Zhongda Hospital, CHINA

## Abstract

**Background:**

Increased incidence of intraoperative awareness was reported in critically ill patients during major operations, particularly under total intravenous (TIVA) or endotracheal general anesthesia (ETGA). However, the incidence and effect of anesthesia techniques on awareness in generally healthy, non-critically ill patients during operations have yet to receive significant attention.

**Methods and results:**

This retrospective matched case-control study was conducted between January 2009 to December 2014. Surgical patients (ASA physical status I-III) whom reported intraoperative awareness during this study period were interviewed and their medical records were reviewed. The potential risk factors for awareness were compared with the non-case matched controls, who were randomly selected from the database. A total of 61436 patients were included and 16 definite cases of intraoperative awareness were identified. Patients who received ETGA and TIVA had significantly higher incidence of developing awareness compared to those who were anesthetized using laryngeal masks (LMA) (P = 0.03). Compared with the matched controls (n = 80), longer anesthesia time was associated with increased incidence of awareness (odds ratio 2.04; 95% CI 1.30–3.20, per hour increase). Perioperative use of muscle relaxant was also associated with increased incidence of awareness, while significantly lower incidence of awareness was found in patients who were anesthetized with volatile anesthetics.

**Conclusions:**

The overall incidence of awareness was 0.023% in the ASA≤ III surgical patients who received general anesthesia. Anesthesia with a laryngeal mask under spontaneous ventilation and supplemented with volatile anesthetics may be the preferred anesthesia technique in generally healthy patients in order to provide a lower risk of intraoperative awareness.

## Introduction

Intraoperative awareness is the unexpected recall of explicit memory during anesthesia. Most patients recall the intraoperative events without pain, some reported vague auditory recall or sensations of dreaming, while a small amount of serious cases experienced pain and tremendous stress [1]. The development of awareness during general anesthesia can be devastating to the patients and anesthesia team. A major concern is that patients whom reported experiencing intraoperative awareness are vulnerable to numerous psychological sequelae after anesthesia, including nightmares, depression, and post-traumatic stress disorder [2,3]. The occurrence of awareness may also implicate medico-legal problems [4], as awareness contributed to 1.9% of the American Society of Anesthesiologists (ASA) Closed Claim Project [5]. The reported incidence of intraoperative awareness varied from 0.0068% to 0.18% [6–8]. More recently, the 5^th^ National Audit Project (NAP5) reported that the incidence of awareness in the United Kingdom was 1 in 19,000 (0.0052%) of anesthesia cases [9]. Historically, patients presenting with higher ASA physical status (ASA PS) and critically ill patients who received cardiothoracic traumatic resuscitation or other major/emergency surgeries are at significantly higher risk of developing intraoperative awareness [10–12], probably due to suboptimal levels of anesthesia during the procedures [13]. It has also been proposed that the use of supraglottic device may mitigate the incidence and severity of awareness [14]. Since no previous studies have characterized the incidence and risk stratification of intraoperative awareness in the generally healthy or less critically ill population, we analyzed the incidence and characterized the associated risk factors for the occurrence of intraoperative awareness in patients with ASA PS I-III during non-critical surgeries under general anesthesia. The primary objective of our study was to identify the risk factors for accidental intraoperative awareness in generally healthy patients, and the second objective was to determine the effects of different anesthesia techniques on the development of awareness during operation.

## Materials and methods

### Patient database

This retrospective chart-review matched case-control study carried out in a tertiary teaching medical center located at Hualien City of Taiwan that consisted of 945 beds. The study was approved by the ethics committee and the institutional review board (IRB, Approval number IRB106-22-B). Since all the data were collected retrospectively from the routine clinical records and questionnaires were completed under the standard practice guidelines, requirement for written informed consent was waived by the committee. In-hospital patients who received anesthesia management for surgical and other medical interventions from January 2009 to December 2014 were visited at bedside within 24 hours after operation. During the routine postoperative visit, nurse anesthetists collected all perioperative adverse events from the patients or caregivers, which including awareness during operation.

### Identification of accidental awareness

Intraoperative awareness is defined as an experience of consciousness under general anesthesia with subsequent recall of the experienced events after emergence [15]. Patients who received regional anesthesia, outpatient surgery, ASA class ≥IV, and those admitted to intensive care units after surgery were excluded from the review. All cases of self-reported intraoperative awareness were interviewed by an experienced nurse anesthetist, and the structured Brice interview was then retrospectively completed with the assistance of the nurse anesthetist [16]. The Brice interview questionnaire included the following five questions:

1. What was the last thing the patient remembered happening before went to asleep?
2. What was the first thing the patient remembered happening on waking?
3. Did the patient dream or have any other experiences whilst he/she was asleep?
4. What was the worst thing about the operation?
5. What was the next worst thing?

A review committee, which consisted of four senior anesthesiologists, was organized. The events and severity of awareness were respectively identified by the review committee using the Michigan awareness instrument and the NAP5 severity [17]. Cases of definite intraoperative awareness had to be approved by all the committee members.

### Matched case-controls

**Fig 1:**
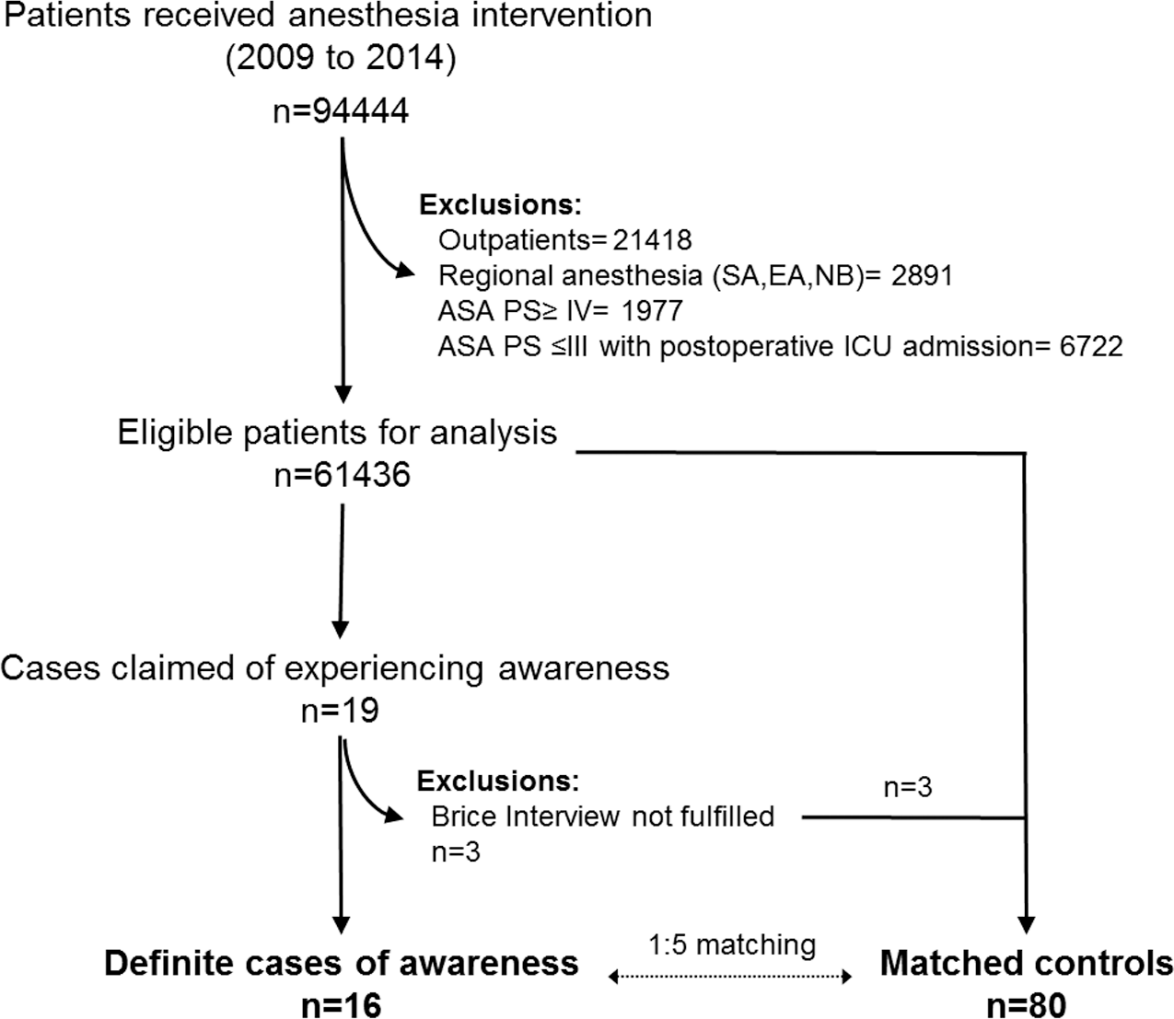
Study design and flow diagram. ASA PS: American Association of Anesthesiologists physical status; EA: epidural anesthesia; ICU: intensive care unit; NB: nerve block; SA: spinal anesthesia.

Control cases were randomly selected from the surgical patients who received general anesthesia without reporting intraoperative awareness during the study period, following exact matching with age, ASA classifications, and gender between the case (awareness) and control (non-awareness) patients in a 1 to 5 ratio (Fig 1). These three characteristic parameters are often used as matching variables because they are generally considered as strong confounders [18].

### Statistics

Since the anesthesia record of one awareness patient was missing from the chart file, the medication of this patient during the perioperative period was not reported. The values of continuous variables were compared using an independent two-sample t test or one-way ANOVA. Categorical variables were compared using chi-square or Fisher’s exact test. The potential risk factors included patient demographic and clinical variables, namely patient age groups, genders, ASA PS, types of anesthesia, duration of anesthesia, anesthetics for maintenance, use of NMBA, use of midazolam, dosage of fentanyl and ephedrine. Conditional logistic regression model was adopted to evaluate the association between these risk factors and intraoperative awareness. Statistical significance was accepted at a level of P< 0.05. All statistical analyses were performed using SAS 9.4 (SAS Institute, Inc., Cary, North Carolina).

## Results

### General outcomes

During the study period, a total of 94444 in-hospital patients received anesthesia management for surgical or other invasive interventions, and of those, 61436 patients were included in this analysis after excluding cases with regional anesthesia, ASA PS≥ IV, or ICU admission after operation (Fig 1). The average annual completion rate (2009–2014) for bedside visiting within 24 h after operation was 97.8% (97.0–98.7%). Nineteen patients claimed to experience awareness during anesthesia, and the Brice interview questionnaire was completed for each patient. After committee review, three patients were excluded from the analysis due to unsustained evidence of intraoperative awareness (Fig 1). The characteristics and detailed descriptions for patients who experienced definite intraoperative awareness with explicit recall were summarized in Table 1.

**Table 1:** Characteristics and descriptions of intraoperative awareness.

Patient ID	Age	Sex	ASA PS	BMI	Surgical procedure	Anesthesia technique	Anesthesia time (h)	Anesthetics	NMBA	Patient’s description about awareness
1	31	F	2	26.8	Debulking operation of cervical cancer	ETGA	6.8	IVGA, Desflurane	Yes	I could hear people talking for a few seconds. I felt some discomfort in my throat and tried to bite the tube. It was very frightening
2	44	F	1	24.8	Laparoscopic uterine myomectomy	ETGA	4.8	Sevoflurane	Yes	My eyes were covered but I could hear them talking. I felt some prodding near my stomach and a bit of pain. It was very frightening
3	22	F	1	20.4	Calf reduction surgery	ETGA	2.6	IVGA	Yes	I could feel something stabbing me for around 2 to 3 minutes and I couldn't move my limbs
4	55	M	2	30.5	Percutaneous nephrolithotomy	ETGA	1.8	IVGA, Sevoflurane	Yes	I woke up during the surgery because of the pain but quickly fell asleep again. I could feel machinery inside of me and it was quite noisy
5	71	F	2	24.1	Radiofrequency ablation of hepatoma	LMA	1.1	IVGA	No	It felt weird that I could hear talking during the surgery
6	68	F	2	26	Partial mastectomy	ETGA	1.4	Sevoflurane	Yes	I could feel things moving around my chest during the surgery, but it didn’t hurt and wasn’t scary
7	54	M	3	23.9	Partial hepatectomy	ETGA	8.3	IVGA	Yes	I could feel being cut on the abdomen but wasn’t able to say it
8	75	M	2	24.7	Laminectomy of lumbar spine	ETGA	5.5	Sevoflurane	Yes	I heard some hammering noises and thought if the anesthetic wasn’t enough. It didn’t hurt
9	51	F	2	24.2	Modified radical mastectomy	ETGA	4.7	IVGA, Sevoflurane	Yes	I felt a sucking kind of pain at the end of the surgery and could faintly hear people talking for a few minutes. It was very scary
10	67	M	2	23.1	Excision of buccal cancer	ETGA	5.8	IVGA	Yes	I could remember people talking and I was so scared
11	74	F	3	27.9	Fusion of lumbar spine	ETGA	3.6	Sevoflurane	Yes	There was hammering but I didn’t know what they were hammering. It didn’t hurt.
12	44	F	3	15.3	Local flap reconstruction	ETGA	0.9	-	-	I felt someone was stitching up the wound
13	74	F	2	25.0	Hemorrhoidectomy	IVGA	0.3	IVGA	No	I was half conscious and could tell people were talking during the surgery
14	45	F	2	17.1	Hemorrhoidectomy	IVGA	0.6	IVGA	No	I could hear the staff talking throughout the surgery, but I didn’t feel anything
15	80	M	3	23.8	Resection of sigmoid cancer	ETGA	3.7	Desflurane	Yes	I could feel a sharp pain from the wound and could feel my abdomen was being operated on
16	52	M	2	24.3	Laminectomy of lumbar spine	ETGA	4.6	Desflurane	Yes	I was awake from when they tried to put me under until they flipped me over

ASA PS: American Society of Anesthesiologists Physical Status; BMI: body mass index; ETGA: endotracheal general anesthesia; IVGA: propofol-based intravenous general anesthesia; NMBA: neuromuscular blocking agents. Types of anesthetics used during operation are not shown in patient #12, as part of the perioperative record was missing.

Female patients were more likely to experience intraoperative awareness than males, but the difference was not statistically significant (62.5% vs 46.2%, P = 0.217; Table 2). Levels of ASA PS (I-III) and age of patients were similar between those with and without awareness (Table 2). In comparison to the non-case patients at risk, significantly fewer patients who developed intraoperative awareness were anesthetized with laryngeal mask anesthesia (LMA; P = 0.003) (Table 2).

**Table 2:** Characteristic analysis of intraoperative awareness in at-risk patients.

Characteristics	Awareness n = 16	No awareness n = 61420	P value
Age (years)	56.5±16.6	52.1±19.2	0.322
Age group (years)			0.511
≤30	1(6.3%)	9072(14.8%)	
30–50	4(25.0%)	17625(28.7%)	
50–70	6(37.5%)	23435(38.2%)	
>70	5(31.3%)	11288(18.4%)	
Gender			0.217
Male	6(37.5%)	33020(53.8%)	
Female	10(62.5%)	28400(46.2%)	
ASA PS			0.735
ASA I-II	12(75.0%)	43714(71.2%)	
ASA III	4(25.0%)	17706(28.8%)	
Types of anesthesia			0.003*
ETGA	13(81.3%)	26728(43.5%)	
IVGA	2(12.5%)	4990(8.1%)	
LMA	1(6.3%)	29702(48.4%)	

ASA PS: American Society of Anesthesiologists Physical Status; ETGA: endotracheal general anesthesia; IVGA: intravenous general anesthesia; LMA: laryngeal mask anesthesia. Data are presented as mean±SD or n (%).

*P< 0.05 is considered as statistically significant.

### Comparison analysis with non-case matched controls

Body weight and level of education were not found to be significantly related to intraoperative awareness, however, the mean duration of anesthesia was found to be significantly longer for the case group (n = 16) compared to the controls (n = 80) (3.5±2.4 vs 1.9±1.2 h, case vs control group; P< 0.001; Table 3). Two patients (12.5%) in the awareness group and 4 patients (5%) in the matched control group expired within 1 year after their last case of anesthesia (P = 0.261; Table 3). The duration between the last case of anesthesia and expiration were 144–301 and 15–341 (range) days in the case and matched control groups respectively. Patient’s education level or personal religious belief was not different between the awareness cases and matched controls (data not shown). The use of propofol-based IVGA, neuromuscular blocking agents and higher doses of fentanyl during operation were associated with significantly higher incidence of developing awareness; while administration of midazolam or doses of ephedrine for treatment of intraoperative hypotension were not different between the awareness and matched control groups (Table 4).

**Table 3:** Characteristic analysis of intraoperative awareness (cases vs matched controls).

Characteristics	Cases n = 16	Matched controls n = 80	P value
Age (years)	56.7±16.6	56.7±16.9	1.000
Age group (years)			1.000
≤30	1(6.3%)	5(6.3%)	
30–50	4(25.0%)	20(25.0%)	
50–70	6(37.5%)	30(37.5%)	
>70	5(31.3%)	25(31.3%)	
Gender			1.000
Male	6(37.5%)	30(37.5%)	
Female	10(62.5%)	50(62.5%)	
ASA PS			1.000
ASA I-II	12(75.0%)	60(75.0%)	
ASA III	4(25.0%)	20(25.0%)	
Types of anesthesia			0.014*
ETGA	13(81.3%)	40(50.0%)	
IVGA	2(12.5%)	4(5.0%)	
LMA	1(6.3%)	36(45.0%)	
BMI	23.9±3.7	25.0±5.2	0.404
BMI group			0.549
18.5–24.9	4(25.0%)	32(40.0%)	
<18.4	2(12.5%)	6(7.5%)	
>25	10(62.5%)	42(52.5%)	
Duration of anesthesia (h)	3.5±2.4	1.9±1.2	<0.001*
Mortality	2(12.5%)	4(5.0%)	0.261

ASA PS: American Society of Anesthesiologists Physiological Status; BMI: body mass index; ETGA: endotracheal general anesthesia; IVGA: intravenous general anesthesia; LMA: laryngeal mask anesthesia. Data are presented as mean±SD or n (%).

*P< 0.05 is considered as statistically significant.

**Table 4:** Anesthetic-related factors for intraoperative awareness (cases vs matched controls).

Anesthetics	Cases n = 15	Matched controls n = 80	P value
Anesthetics for maintenance			<0.001*
Propofol-based IVGA	6(40%)	10(12.5%)	
Isoflurane/sevoflurane/desflurane	6(40%)	68(85.0%)	
Combined anesthetics	3(20%)	2(2.5%)	
Use of NMBA			0.047*
Yes	12(80%)	40(50%)	
No	3(20%)	40(50%)	
Use of midazolam			0.110
Yes	3(20%)	5(6.3%)	
No	12(80%)	75(93.7%)	
Dose of fentanyl (μg)	211±162	101±62	<0.001*
Dose of ephedrine (mg)	3.6±8.6	5.2±9.0	0.533

IVGA: intravenous general anesthesia; NMBA: neuromuscular blocking agents. The original anesthesia record of a patient (#12) in the case group was missing, drugs administration during intraoperative period was not reported in this patient. Data are presented as mean±SD or n (%).

*P< 0.05 is considered as statistically significant.

### Conditional logistic regression analysis

The crude odds ratios of experiencing intraoperative awareness were 11.75 (95% CI 1.47–93.74; P = 0.020) and 17.75 (95% CI 1.35–233.96; P = 0.028) respectively for patients who received ETGA/IVG and LMA, but the differences became insignificant after multivariate analysis (Table 5). The effects of anesthetics and other medications during perioperative period were also analyzed. Anesthesia with propofol-based IVGA and use of muscle relaxants during the operation was associated with significantly higher incidence of intraoperative awareness compared to volatile anesthetics and those received no muscle relaxation, respectively (Table 5). Perioperative use of midazolam and fentanyl did not affect the incidence of awareness in multivariate analysis. Administration of ephedrine for correction of hypotension during operation did not affect the development of awareness, but prolonged duration of anesthesia significantly increased the risk of awareness (AOR 4.26 per hour increase; 95% CI 1.33–13.63; P = 0.015).

**Table 5:** Conditional logistic regression analysis of the risk factors associated with awareness.

Characteristics	Control		Case		Crude OR	95% CI	P	AOR	95% CI	P
	n	%	n	%						
Type of anesthesia										
ETGA	40	50.0	13	81.3	11.75	1.5, 93.7	0.020*	<0.01	<0.01, 2.6	0.091
IVGA	4	5.0	2	12.5	17.75	1.4,234.0	0.028*	3.13	0.05, 193.8	0.588
LMA	36	45.0	1	6.3	Ref			Ref		
Use of neuromuscular blocking agents										
No	40	50.0	12	20.0	Ref			Ref		
Yes	40	50.0	3	80.0	4.64	1.2, 18.0	0.027*	>999.9	1.25, >999.9	0.044*
Maintenance of anesthesia										
Propofol-based IVGA	10	12.5	6	40.0	Ref			Ref		
Volatile anesthetics	68	85.0	6	40.0	0.16	0.04, 0.6	0.006*	0.03	<0.01, 0.9	0.041*
Combined	2	2.5	3	20.0	2.36	0.4, 15.9	0.378	0.32	<0.01, 24.5	0.605
Use of midazolam										
Yes	75	93.7	12	80.0	Ref					
No	5	6.3	3	20.0	3.75	0.8, 16.8	0.084	1.80	0.06, 50.5	0.731
Fentanyl (μg)					1.01	1.00, 1.02	0.005*	0.99	0.98, 1.00	0.170
Ephedrine (mg)					0.97	0.9, 1.1	0.498	0.89	0.78, 1.02	0.102
Duration of anesthesia (h)					2.04	1.3, 3.2	0.002*	4.26	1.3, 13.6	0.015*

OR: odd ratio; AOR: adjusted odd ratio; CI: confidence interval; ETGA: endotracheal general anesthesia; IVGA: intravenous general anesthesia; LMA: laryngeal mask anesthesia; Ref: reference. The original anesthesia record of a patient (#12) in the case group was missing, drug administration (neuromuscular blocking agent, maintenance anesthetic, midazolam, fentanyl and ephedrine) during intraoperative period was not reported in this patient.

*P< 0.05 is considered as statistically significant.

## Discussion

By analyzing our post-anesthesia quality assurance records through 2009–2014, we determined the overall incidence of developing intraoperative awareness among surgical patients with ASA PS I-III to be 0.023%. General anesthesia using endotracheal tubes, propofol-based intravenous anesthesia techniques, and prolonged periods under anesthesia were especially associated with increased risk for developing awareness. Clinical research regarding the incidence and identification of the associated risk factors in accidental intraoperative awareness has been a challenge to epidemiologic studies, as intraoperative awareness is a relatively rare perioperative event and subject to high levels of discrepancy in clinical diagnostic criteria [19]. The reported incidence of intraoperative awareness was generally higher in prospective studies (1:250 to 1:1000) [6,7,20,21], which may have been confounded by false memories or dreaming [19]. In large-scale retrospective studies, the incidence of awareness was significantly lower (1:10000 to 1:19000) in general the surgical population and cancer patients [8,22,23]. It is clear that retrospective studies are subject to under-reporting, missing cases, and recall bias. A total of 19 patients claimed to awake during general anesthesia in our study, but 3 patients without explicit recall were excluded from the final analysis, as the review committee members believed that they were more likely to have perioperative dreaming rather than awareness [24]. Therefore, we identified an overall incidence of 0.023% (approximately 1:4300) for developing accidental intraoperative awareness in the generally healthy surgical patients (ASA PS class ≤ 3), which falls between the incidence reported in the prospective and retrospective studies. Nevertheless, we believed that the overall incidence of our study could actually be higher, as the critically ill patients and those who transferred to ICU after major operations were excluded from the analysis.

There is sufficient clinical evidence to suggest that patients presenting with high ASA PS (> III) and those admitted to ICU for postoperative care are at significantly higher risk of awareness [1,3,8]. Since high risk patients (ASA IV-V) contribute to only a limited portion (<10%) of the entire surgical population [25,26], the exclusion of these high-risk patients or major surgery (such as cardiac bypass or trauma resuscitation surgery) from our post-anesthesia quality assurance record may provide better insight into the other risk factors associated with intraoperative awareness in the majority population.

In patients with lower anesthesia risks, the class of ASA PS did not affect the occurrence of awareness. Age was also not a significant differentiating factor for patients who developed awareness and those who did not. Female gender has been considered a potential risk factor for awareness, as females are quicker to emerge from anesthesia [27–29]. Our study showed that the proportion of female patients (62.5% vs 37.5%) was higher in the awareness group, even though there were more male patients included in the whole study population (53.8% vs 46.2%). Nonetheless, the difference in genders was not statistically significant (P = 0.217). In light of the underpowered statistical analysis, we suggest that gender should not be excluded as a risk factor for intraoperative awareness until more substantial clinical evidence is found.

The other potential associated risk factors were determined by comparing the characteristics and intraoperative parameters between the case (n = 16) and matched non-case controls (n = 80). Previous studies have reported a higher incidence of awareness in obese patients [22,30]. Our analysis showed that body mass index (BMI) was not an independent factor for awareness in patients with ASA PS I-III after case-control matching. Most interestingly, we found that total anesthesia time was significantly longer in patients who eventually developed awareness with a crude odd ratio of 2.04 (95% CI 1.30–3.20) per hour increase of anesthesia time. The effect of anesthesia duration on the development of intraoperative awareness has not been previously reported. In our opinion, chances of medication or equipment errors are more likely to happen during prolonged operations and gaps between working shifts [31].

Anesthesia-related medications, such as anesthetics, muscle relaxants and opioids, have been extensively reported to affect the incidence of intraoperative awareness [20,22]. In line with these previous studies, our study confirmed that anesthetized with volatile halogenated anesthetics was associated with significantly lower incidence of awareness than propofol-based IVGA. Minimal alveolar concentrations (MAC) of volatile anesthetics and the processed electroencephalographic bispectral index (BIS) are the two commonly recommended techniques in the monitoring of depth of anesthesia during operation to avoid anesthesia awareness [32]. In our institute, MAC is routinely monitored throughout the anesthesia period whenever an inhaled anesthetic is administered, but BIS monitoring for propofol-based IVGA is rarely used, as the accessories of the BIS system are not subsidized by the Taiwan National Health Insurance. In fact, the application of processed electroencephalogram (including BIS, E-Entropy, or Narcotrend) during general anesthesia is as low as the 2.8% reported in the United Kingdom [25]. Although our results were not able to establish a direct causal relationship, it is clinically reasonable to apply processed electroencephalogram for monitoring the depth of anesthesia, particularly during propofol-based IVGA, in order to reduce the occurrence of awareness [33].

One of the most important risk factors for intraoperative awareness is the use of neuromuscular blocking agents [22]. Loss of reflexive somatic responses to surgical stimuli following profound motor paralysis diminished the postural signs of awareness in patients who received general anesthesia [29]. Consistent with these previous studies, our study demonstrates that use of neuromuscular blocking agent significantly increased awareness events by an odds ratio of 4.64 (95% CI 1.19–18.04). The dosage of a short-acting opioid fentanyl administered during operation was also found to be positively correlated with the occurrence of awareness (OR 1.01 per μg increase, 95% CI 1.00, 1.02; P = 0.005). Since the total doses of fentanyl administrated during operation are inevitably increased for lengthier surgical procedures, doses of fentanyl might be a dependent variable of prolonged anesthesia time. On the other hand, the perioperative use of midazolam did not significantly affect the incidence of awareness.

Since light anesthesia secondary to perioperative hypotension has been recognized as a crucial risk factor for awareness [34], we compared the dosage of ephedrine (the most commonly used inotropic agent during anesthesia in Taiwan) between the case and non-case matched controls as a surrogate indicator for perioperative hypotension. Our results showed that the doses of ephedrine used during operation periods were similar between the two groups. However, without appropriate comparison of hemodynamic measurements, the actual effects of perioperative hypotension on the development of awareness in non-critically ill surgical patients have yet to reach a conclusion. In this study, we also observed that the 1-year mortality rate after the last case of anesthesia was increased in patients who developed intraoperative awareness (12.5% vs 5%, case vs matched control; P = 0.129). Since the numbers of postanesthesia mortality were too small and the time from the last case of anesthesia to the date of expiration ranged from 15 to 341 days, analysis of a larger scale database or prospective studies are warranted to determine the actual clinical implication of intraoperative awareness and early postoperative mortality.

The most important finding of analysis in this case-control matching study is that the occurrence of intraoperative awareness was significantly reduced in patients who received LMA compared to those that received ETGA and IVGA. After matching for age, gender, and ASA PS, the use of LMA was found to be associated with significantly reduced risk of developing intraoperative awareness compared to the use of ETGA and IVGA. Compared with LMA, ETGA and IVGA carried crude odds ratios of 11.75 (95% CI 1.47–93.74) and 17.75 (95% CI 1.35–233.98) respectively for intraoperative awareness.

Based on our analysis, we tried to address the important issue that whether different types of anesthetic techniques would affect the incidence of intraoperative awareness. The use of LMA supplemented by volatile anesthetics has become a widely used technique that can help avoid the administration of neuromuscular agents throughout the anesthetic period. The uptake of inhaled anesthetics depends on the patient’s spontaneous ventilation with constant monitoring of anesthetic depth by MAC levels. Since LMA is the preferred technique for the majority of less invasive surgical procedures in all age groups [35–36], the results of our study support the ratiocination proposed by Drs. Avidan and Mashour that the use of supraglottis device anesthesia would mitigate both the incidence and severity of intraoperative awareness [14].

There are a number of limitations in our study. First, the retrospective study design limits our ability to establish a direct causal relationship between the measured variables and intraoperative awareness incidence. The study design is also subject to missing cases and memory loss, as awareness is not the primary parameter in our post-anesthesia quality assurance record. Hence, the results of our study might be prone to recall bias and confounded by other unmeasured risk factors. Second, although high completion rates of postoperative visits (98%) might provide sufficient power in detecting intraoperative awareness in the in-hospital surgical patients, the incidence of awareness in outpatient ambulatory surgery was not included in the analysis. Since all outpatients are cared for at least 1 hour after recovery from anesthesia in our hospital, any major postanesthesia complications should have been recorded by the staff in the post-anesthesia care unit. Third, the numbers of definite awareness reported in this study were relatively small, which made certain statistical analysis underpowered, in particular the gender and 1-year mortality analyses. However, considering the fact that intraoperative awareness is a rare event, the collection of large sample size of awareness is very strenuous in the retrospective, or even in the prospective studies [7,8,20]. Finally, patients with perioperative awareness were not routinely followed up after discharge from our hospital. Therefore, we are not able to present the long-term psychophysiological consequences of these patients.

In this study we determined the overall incidence of developing intraoperative awareness among non-critically ill patients to be 0.023%. General anesthesia using endotracheal tubes, intravenous propofol-based techniques, and prolonged periods under anesthesia were especially associated with increased risk for developing awareness. The use of neuromuscular blocking agents during anesthesia also carried significantly higher incidence of awareness. Base on these findings, we suggest that spontaneous ventilation via a laryngeal mask supplemented with volatile anesthetics may a preferable anesthesia technique in order to provide a lower risk of intraoperative awareness in generally healthy patients.

## Supporting information

S1 Table Minimal dataset for statistical analysis. (PDF)

S2 Table Codebook for minimal dataset. (PDF)
