# Supplementary material for: Inhalation of volatile anesthetics via a laryngeal mask is associated with lower incidence of intraoperative awareness in non-critically ill patients
Source: PLoS One. 2017 Oct 26;12(10):e0186337. doi: 10.1371/journal.pone.0186337 (PMC5658000; doi:10.1371/journal.pone.0186337)
Supplement: S1 Table — (PDF) [file pone.0186337.s001.pdf]

Variable	Description	Coding					
		0	1	2	3	4	Remark
Group	To distinguish whether a patient occurred intraoperative awareness	No	Yes				
No	Match pair id						
Year	OP year						
Gender	Gender		Male	Female			
Age	Patient's age on OP date						
Age_Group	Age Group(divided into 4 groups)		≦30 y/o	30-50 y/o	50-70 y/o	>70 y/o	
ASA	ASA						
ASA_Group	ASA Group(divided into 2 groups)		1~2	3			
ASA_Type	Anesthesia Type		ETGA	IVGA	LMA		
BMI	BMI						
BMI_Group	BMI Group(divided into 3 groups)		Normal	Underweight	Overweight or Obese		
ASA_Hour	Duration of anesthesia(hour)						
Expired	Expired		No	Yes			
Fentanyl	Fentanyl amount(ug)						
Midazolam	Use of Midazolam		No	Yes			
Ephedrine	Ephedrine amount(mg)						
NMBA	Use of NMBA		No	Yes			
Maintenance	Maintenance of anesthesia		Propofol-based IVGA	Volatile anesthetics	Combined		
