# Supplementary material for: Inhalation of volatile anesthetics via a laryngeal mask is associated with lower incidence of intraoperative awareness in non-critically ill patients
Source: PLoS One. 2017 Oct 26;12(10):e0186337. doi: 10.1371/journal.pone.0186337 (PMC5658000; doi:10.1371/journal.pone.0186337)
Supplement: S2 Table — (PDF) [file pone.0186337.s002.pdf]

Group	No	Year	Gender	Age	Age_Group	ASA	ASA_Group	ASA_Type	BMI	BMI_Group
1	1	2014	2	31	2	2	1	1	26.8	3
1	2	2014	2	44	2	1	1	1	24.8	3
1	3	2014	2	22	1	1	1	1	20.4	1
1	4	2013	1	55	3	2	1	1	30.5	3
1	5	2013	2	71	4	2	1	3	24.1	3
1	6	2012	2	68	3	2	1	1	26.0	3
1	7	2011	1	54	3	3	2	1	23.9	1
1	8	2011	1	75	4	2	1	1	24.7	3
1	9	2011	2	51	3	2	1	1	24.2	3
1	10	2011	1	67	3	2	1	1	23.1	1
1	11	2011	2	74	4	3	2	1	27.9	3
1	12	2011	2	44	2	3	2	1	15.3	2
1	13	2009	2	74	4	2	1	2	25.0	3
1	14	2009	2	45	2	2	1	2	17.1	2
1	15	2009	1	80	4	3	2	1	23.8	1
1	16	2009	1	52	3	2	1	1	24.3	3
0	1	2009	2	31	2	2	1	1	23.1	1
0	1	2010	2	31	2	2	1	3	28.8	3
0	1	2011	2	31	2	2	1	1	21.4	1
0	1	2010	2	31	2	2	1	1	19.8	1
0	1	2012	2	31	2	2	1	3	25.1	3
0	2	2010	2	44	2	1	1	1	22.8	1
0	2	2010	2	44	2	1	1	2	31.0	3
0	2	2009	2	44	2	1	1	1	23.0	1
0	2	2010	2	44	2	1	1	1	21.5	1
0	2	2010	2	44	2	1	1	1	26.0	3
0	3	2012	2	22	1	1	1	1	21.5	1
0	3	2013	2	22	1	1	1	3	17.1	2
0	3	2010	2	22	1	1	1	3	17.1	2
0	3	2011	2	22	1	1	1	1	18.4	2
0	3	2010	2	22	1	1	1	1	19.2	1
0	4	2010	1	55	3	2	1	1	28.1	3
0	4	2009	1	55	3	2	1	2	19.1	1
0	4	2010	1	55	3	2	1	3	23.3	1
0	4	2011	1	55	3	2	1	3	20.6	1
0	4	2011	1	55	3	2	1	1	36.4	3
0	5	2011	2	71	4	2	1	1	27.8	3
0	5	2009	2	71	4	2	1	1	25.4	3
0	5	2013	2	71	4	2	1	1	27.5	3
0	5	2009	2	71	4	2	1	3	24.8	3
0	5	2013	2	71	4	2	1	3	27.8	3
0	6	2010	2	68	3	2	1	3	33.2	3
0	6	2010	2	68	3	2	1	3	27.1	3
0	6	2012	2	68	3	2	1	1	30.7	3
0	6	2009	2	68	3	2	1	3	25.8	3
0	6	2011	2	68	3	2	1	1	26.4	3
0	7	2010	1	54	3	3	2	3	25.0	3
0	7	2009	1	54	3	3	2	1	23.7	1
0	7	2011	1	54	3	3	2	1	14.7	2
0	7	2010	1	54	3	3	2	1	25.6	3
0	7	2013	1	54	3	3	2	3	25.9	3
0	8	2010	1	75	4	2	1	1	27.6	3
0	8	2009	1	75	4	2	1	3	27.9	3
0	8	2011	1	75	4	2	1	3	27.0	3
0	8	2013	1	75	4	2	1	2	23.4	1
0	8	2012	1	75	4	2	1	1	27.2	3

0	9	2013	2	51	3	2	1	3	27.7	3
0	9	2012	2	51	3	2	1	1	22.6	1
0	9	2012	2	51	3	2	1	3	32.9	3
0	9	2009	2	51	3	2	1	3	23.5	1
0	9	2010	2	51	3	2	1	3	28.6	3
0	10	2014	1	67	3	2	1	1	18.3	2
0	10	2013	1	67	3	2	1	1	27.0	3
0	10	2009	1	67	3	2	1	3	25.4	3
0	10	2010	1	67	3	2	1	1	22.5	1
0	10	2011	1	67	3	2	1	3	23.2	1
0	11	2013	2	74	4	3	2	1	20.4	1
0	11	2011	2	74	4	3	2	3	23.0	1
0	11	2011	2	74	4	3	2	2	31.2	3
0	11	2012	2	74	4	3	2	1	21.7	1
0	11	2012	2	74	4	3	2	3	30.6	3
0	12	2011	2	44	2	3	2	1	23.7	1
0	12	2009	2	44	2	3	2	1	18.2	2
0	12	2010	2	44	2	3	2	1	22.0	1
0	12	2012	2	44	2	3	2	1	19.9	1
0	12	2012	2	44	2	3	2	1	21.1	1
0	13	2013	2	74	4	2	1	3	29.6	3
0	13	2011	2	74	4	2	1	1	30.6	3
0	13	2012	2	74	4	2	1	3	26.6	3
0	13	2013	2	74	4	2	1	3	29.8	3
0	13	2010	2	74	4	2	1	3	18.8	1
0	14	2012	2	45	2	2	1	1	51.9	3
0	14	2011	2	45	2	2	1	1	24.0	1
0	14	2011	2	45	2	2	1	1	29.6	3
0	14	2013	2	45	2	2	1	3	26.7	3
0	14	2011	2	45	2	2	1	1	24.6	3
0	15	2009	1	80	4	3	2	3	20.0	1
0	15	2011	1	80	4	3	2	3	25.4	3
0	15	2013	1	80	4	3	2	1	30.6	3
0	15	2012	1	80	4	3	2	3	23.3	1
0	15	2013	1	80	4	3	2	3	19.6	1
0	16	2009	1	52	3	2	1	3	23.9	1
0	16	2011	1	52	3	2	1	1	24.2	3
0	16	2009	1	52	3	2	1	3	22.8	1
0	16	2012	1	52	3	2	1	3	25.8	3
0	16	2012	1	52	3	2	1	3	23.0	1

ASA_Hour	Expired	Fentanyl	Midazolam	Ephedrine	NMBA	Maintenance
6.8	0	300	0	0	1	3
4.8	0	150	0	0	1	2
2.6	0	200	0	0	1	1
1.8	0	150	0	0	1	3
1.1	0	75	0	0	0	1
1.4	0	100	0	0	1	2
8.3	1	650	0	0	1	1
5.5	0	100	0	8	1	2
4.7	0	350	1	0	1	3
5.8	1	450	0	0	1	1
3.6	0	150	0	30	1	2
0.9	0					
0.3	0	100	0	0	0	1
0.6	0	100	0	16	1	2
3.7	0	200	1	0	1	2
4.6	0	100	1	0	0	1
2.7	0	300	0	0	1	1
1.8	0	0	0	0	0	2
1.9	0	100	0	0	1	2
3.3	0	100	0	0	1	2
0.7	0	50	0	0	0	2
3.4	0	100	0	0	1	2
0.8	0	100	1	0	0	1
3.5	0	150	0	0	1	2
1.8	0	100	0	6	1	2
1.5	0	100	0	0	1	2
2.0	0	100	0	16	1	2
1.4	0	100	0	0	0	2
3.1	0	175	0	0	0	3
1.0	0	100	0	0	1	2
3.4	0	200	0	0	1	1
2.3	0	100	0	12	1	2
0.3	0	100	1	0	0	1
0.5	0	50	0	0	0	2
0.8	0	75	0	0	0	2
1.6	0	175	0	16	1	2
0.7	0	100	0	0	1	2
1.2	0	100	0	0	1	2
3.1	0	200	0	0	1	2
1.6	0	50	0	0	0	2
1.8	0	150	0	0	0	2
2.3	0	50	0	24	0	2
2.5	0	75	0	12	0	2
2.1	0	100	0	8	1	2
0.5	0	25	0	14	0	2
1.0	0	100	0	0	1	2
1.0	0	50	0	44	0	2
7.0	0	150	1	18	1	2
1.5	1	100	0	0	1	1
2.3	0	300	0	0	1	2
0.6	0	75	0	0	0	2
0.7	0	50	0	0	1	2
0.9	0	50	0	0	0	2
2.7	0	125	0	16	0	2
1.4	0	100	1	0	0	1
5.2	0	250	0	18	1	1

1.9	0	175	0	0	0	2
2.3	0	100	0	0	1	2
0.6	0	50	0	0	0	2
0.5	0	50	0	0	0	2
2.3	0	75	0	0	0	2
3.9	0	100	0	10	1	2
2.5	0	150	0	0	1	2
0.8	0	50	0	0	0	2
1.1	0	100	0	0	1	2
3.0	0	100	0	16	0	2
3.2	0	100	0	32	1	2
1.0	0	50	0	0	0	2
0.9	0	0	0	4	0	1
3.5	1	150	0	6	1	2
0.2	0	50	0	0	0	2
1.0	0	100	0	0	1	2
1.7	0	100	0	0	1	2
1.8	0	0	1	0	1	1
1.3	0	100	0	16	1	2
1.5	0	100	0	0	1	2
2.6	0	100	0	12	0	2
4.7	0	325	0	36	1	3
0.5	0	50	0	0	0	2
1.3	0	100	0	8	0	2
3.8	0	50	0	8	0	2
1.5	0	100	0	0	1	2
2.1	0	100	0	0	1	2
3.3	0	175	0	0	1	2
0.8	0	75	0	0	0	2
0.8	0	100	0	0	1	1
2.2	0	25	0	0	0	2
1.8	1	50	0	4	0	2
2.2	0	150	0	0	1	2
2.2	0	50	0	6	0	2
0.6	1	50	0	18	0	2
1.8	0	50	0	14	0	2
2.2	0	150	0	8	1	2
0.8	0	100	0	0	0	2
1.1	0	50	0	0	0	2
1.1	0	100	0	12	0	2
